# *ZED1-related kinase 13* is required for resistance against *Pseudoidium neolycopersici* in Arabidopsis accession Bla-6

**DOI:** 10.3389/fpls.2023.1111322

**Published:** 2023-03-21

**Authors:** Miguel I. Santillán Martínez, Dongli Gao, Michela Appiano, Inge Derks, Robin P. Huibers, Glenn Spil, Xulan Wang, Richard G. F. Visser, Anne-Marie A. Wolters, Yuling Bai

**Affiliations:** Plant Breeding, Wageningen University & Research, Wageningen, Netherlands

**Keywords:** powdery mildew, resistance, Arabidopsis, ZRK13, CRISPR/Cas9

## Abstract

To explore specific components of resistance against the tomato-adapted powdery mildew pathogen *Pseudoidium neolycopersici* (*On*) in the model plant Arabidopsis, we performed a disease assay in 123 accessions. When testing the resistance in the F_1_ from crossings between resistant accessions with susceptible Col-0 or Sha, only the progeny of the cross between accession Bla-6 and Col-0 displayed a completely resistant phenotype. The resistance in Bla-6 is known to be specific for *Pseudoidium neolycopersici*. QTL analysis and fine-mapping through several rounds of recombinant screenings allowed us to locate a major resistance QTL in an interval on chromosome 1, containing two candidate genes and an intergenic insertion. *Via* CRISPR/Cas9 targeted mutagenesis, we could show that knocking out the *ZED-1 RELATED KINASE 13* (*ZRK13*) gene compromised the *On* resistance in Bla-6. Several polymorphisms are observed in the ZRK13 allelic variant of Bla-6 when compared to the Col-0 protein.

## Introduction

Powdery mildew (PM) is one of the most widely occurring plant diseases in the world, observed in almost 10,000 different plant species, including several economically important crops (Takamatsu, 2004; Glawe, 2008). Pathogens causing PM are a diverse group of more than 400 species of fungi of the *Erysiphales* order (Takamatsu, 2004). PMs establish an obligate biotrophic interaction with their hosts, thus depending on living plant cells to complete their life cycles (Glawe, 2008). To defend themselves, plants have evolved active resistance mechanisms that rely on two interconnected layers of defense (Jones and Dangl, 2006; Dodds and Rathjen, 2010; Ngou et al., 2022a; Ngou et al., 2022b). The first layer is activated upon the recognition of pathogen-associated molecular patterns (PAMPs) by pattern recognition receptors (PRRs), located at the plant cell surface, and leading to PAMP-triggered immunity (PTI). The second layer is effector-triggered immunity (ETI) that is activated upon recognition of pathogen effectors by the plant’s resistance (R) proteins. A faster and stronger defense response is produced in ETI compared to PTI, such as a hypersensitive response (HR), a fast and localized type of cell death.

The model plant *Arabidopsis thaliana* has been an important resource to study plant-pathogen interactions. Specifically, the Arabidopsis-PM pathosystem has been useful to identify and characterize host resistance mechanisms (Kuhn et al., 2016). Arabidopsis is known to be infected by four PM species, namely *Erisyphe cruciferarum* (Koch and Slusarenko, 1990), *Golovinomyces* (syn. *Erysiphe*) *cichoracearum* (Adam and Somerville, 1996), *Golovinomyces* (syn. *Erysiphe*) *orontii* (Plotnikova et al., 1998), and the tomato PM pathogen *Pseudoidium* (syn. *Oidium*) *neolycopersici* (*On*) (Xiao et al., 2001). Resistance to PM in Arabidopsis has been shown to be predominantly polygenic (Göllner et al., 2008) and to date, the only dominantly-inherited *R* locus against PM in Arabidopsis is *RPW8*. This locus is located on chromosome 3 and contains two paralogs, *RPW8.1* and *RPW8.2* in accession Ms-0 (Xiao et al., 2001). Interestingly, the proteins encoded by these genes share only a limited homology to other Nucleotide-Binding Leucine-Rich Repeats (NB-LRR)-like R proteins but are able to signal through components used by other NB-LRRs, such as the activation of the salicylic acid-dependent pathways (Xiao et al., 2003; Xiao et al., 2005; Wang et al., 2009). The resistance conferred by *RPW8* is known to be broad-spectrum (Xiao et al., 2001, 2003). However, when testing several accessions of Arabidopsis carrying allelic variants of the *RPW8* paralogs, not all accessions were resistant to the tomato PM pathogen *On*, while being resistant to the other three PM pathogens known to infect Arabidopsis (Göllner et al., 2008). In particular, accession Sha, which harbors the functional allele of *RPW8* and is fully resistant against *E. cruciferarum*, *G. cichoracearum* and *G. orontii*, is susceptible to *On* (Göllner et al., 2008). Additionally, heterologous expression of *RPW8* genes in tomato failed to confer enhanced resistance against *On* (Xiao et al., 2003). This may imply that unique genetic factors in Arabidopsis are contributing to resistance to *On*. The Arabidopsis-*On* interaction has been less extensively characterized compared to that between Arabidopsis and the other three PM species. Therefore, studying this biotrophic relationship may provide new insights to unravel differential genetic components of resistance and susceptibility to *On*.

With the objective of further exploring the resistance in Arabidopsis against *On*, we previously performed a disease assay on 123 Arabidopsis accessions, of which 40 showed to be fully resistant. Further studies of these accessions allowed us to identify a natural mutation of *EDR1* conferring resistance to *On* in accession C24 (Gao et al., 2015). In the present study we describe the mapping and identification of the gene responsible for the resistance against *On* observed in accession Bla-6. Previously, Adam et al. (1999) reported that Bla-6 was susceptible to *G. cichoracearum* and *E. cruciferarum* and thus does not carry the functional *RPW8* gene. We report the finding of a novel dominant resistance gene in Arabidopsis accession Bla-6 against *On*. To our knowledge, this is the first Arabidopsis dominant PM resistance to be characterized through its interaction with *On*. We fine-mapped the candidate region through several rounds of recombinant screenings and used CRISPR/Cas9 targeting the candidate genes to elucidate the gene responsible for the resistance.

## Materials and methods

### Plant material, growth conditions and disease assays

Arabidopsis accessions Bla-6 (accession ID 6621) and Col-0 were obtained from the Max Planck Institute in Köln, Germany. The Arabidopsis plants were grown in soil substrate in a growing chamber with day/night cycles of 16h/8h and 10h/14h in a temperature of 21°C and relative humidity of 70%. For the disease assays, 3 to 4-week old plants were inoculated by spraying a conidiospore suspension (2.5 to 5.8 × 10^5^ spores/mL) of the Wageningen isolate of *On*, which was maintained on tomato *cv* Moneymaker plants. The disease symptoms were scored visually at 8-12 days post inoculation with a score from 0 to 3 (0, no sporulation; 1, slight sporulation; 2 moderate sporulation; 3 abundant sporulation; **Supplementary Figure 1**) for the QTL mapping and with a qualitative score of R (resistant) or S (susceptible) for the fine mapping and mutant screening.

### Mapping, PCR-based sequencing, and analysis of sequencing data

A preliminary QTL analysis in the F_2_ was performed using Indel markers RH555/556, RH473/474, RH565/566, RH481/482 and RH569/570 (**Supplementary Table 1**), based on genome sequence differences between accessions Columbia (Col-0) and Landsberg erecta (Ler) (Hou et al., 2010), using Joinmap4 (van Ooijen, 2006) and MapQTL6 (van Ooijen, 2009). Additional Indel markers were tested for polymorphisms between accessions Col-0 and Bla-6. SNP markers for mapping were developed based on PCR sequencing results. CAPS markers M24, M36, M6 and M14 (**Supplementary Table 2**) were developed based on polymorphisms between Col-0 and Bla-6 retrieved from the Gramene SNP query database (https://archive.gramene.org/db/diversity/snp_query). Primers for PCR-based sequencing for fine mapping and sequencing of the candidate region (**Supplementary Table 2**) were designed based on the TAIR reference genome of Col-0 (www.arabidopsis.org).

Analysis and visualization of sequences were done using SnapGene (www.snapgene.com). Sequence alignments were created using MAFFT version 7 (https://mafft.cbrc.jp/alignment/server/). Predictions of potential genes were retrieved from FGENESH (http://www.softberry.com). ORFfinder (https://www.ncbi.nlm.nih.gov/orffinder/) was used to search for open reading frames. Protein information was retrieved from Uniprot (https://www.uniprot.org/).

### CRISPR/Cas9 targeted mutagenesis

PCR-based sequencing was used to obtain the sequence of the candidate region using the primers described in **Supplementary Table 2**. A set of four single guide RNAs (sgRNAs) was designed to target each of the three candidate loci in the candidate region (**Supplementary Table 3**). All sgRNAs were designed using the CRISPOR (Concordet and Haeussler, 2018) and CC-Top (Stemmer et al., 2015) webtools. G + C content of the sgRNAs was calculated using the ENDMEMO webtool (http://www.endmemo.com/bio/gc.php) and folding of the gRNAs was predicted using the Mfold web server (Zuker, 2003). The sgRNAs were selected following the criteria described by (Liang et al., 2016) and assessing the efficiency (CCTop, Wu-Crispr, Gpp and Doench16) and specificity (MIT specificity score, CRISPRater efficacy score) provided by CRISPOR and CC-Top.

CRISPR/Cas9 constructs for transformation were built in two steps (levels) *via* the Golden Gate cloning system (Weber et al., 2011; Engler and Marillonnet, 2014). Level 0 constructs contained promoters, 5’ untranslated regions, coding sequences, signal peptides and terminators. Level 1 constructs contained complete gene expression units which were later transferred to the level 2 construct, carrying the multiple gene expression unit (Weber et al., 2011). The *Cas9* gene was driven by the Arabidopsis AtUBI10 promoter from ubiquitin gene At4g05320 (Castel et al., 2019; **Supplementary Table 4**). Each of the sgRNAs were ligated to specific backbones of the CRISPR-Pink system vectors, provided by Mark Youles (TSL Norwich, Synbio, **Supplementary Table 4**). Cloning of level 1 and 2 constructs was done using *E. coli* DH5α chemically competent cells. The final level 2 CRISPR constructs were transformed to *Agrobacterium* strain AGL1 + virG. The transformation construct included FAST-Red as a marker to optimize the selection of transformed seeds.

Arabidopsis Bla-6 plants were transformed using a modified method of flower dipping (Clough and Bent, 1998). Instead of dipping floral buds, drops of the infiltration medium [sucrose (50g/L) and Silwet L-77 (200µl/L)] containing *Agrobacterium* carrying the level 2 CRISPR construct were deposited onto unopened flower buds in two events within seven days. After floral dripping transformation, T_1_ seeds were harvested and selected based on the FAST Red fluorescence using a binocular microscope. The seeds were subsequently stratified to break dormancy before being sown.

The TIDE webtool (Brinkman et al., 2014) was used to detect sequence trace decomposition in sequencing data of mutants.

## Results

### Resistance in Bla-6 is governed by a dominant locus on chromosome 1

**Figure 1:**
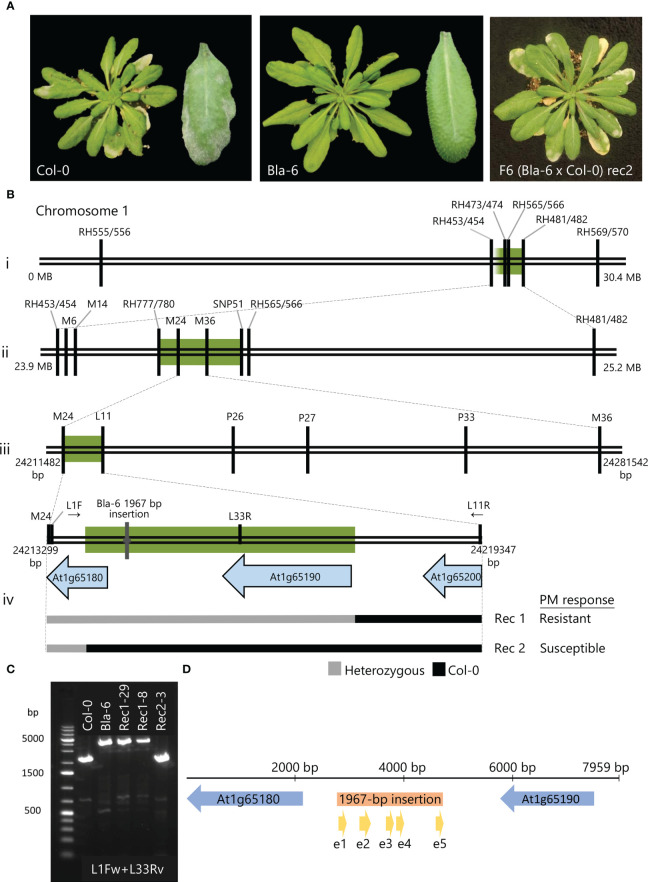
Fine-mapping of the resistance to *Pseudoidium neolycopersici* (*On*) in Arabidopsis accession Bla-6. **(A)** Disease response of Col-0 and Bla-6 to *On* at 12 days post-inoculation (12dpi): heavy sporulation over the infected leaves of susceptible accession Col-0 and lack of visible symptoms in accession Bla-6. *On* disease symptoms on F6 (Bla-6 x Col-0) progeny from susceptible recombinant Rec2. **(B)** Location of markers for fine-mapping of the resistance locus in chromosome 1: green bars indicate the candidate regions for each of the three recombinant screenings (i to iii) and PCR-based sequencing of F5 informative recombinants Rec1 and Rec2 with contrasting phenotypic response upon *On* inoculation (iv). **(C)** Agarose gel electrophoresis of the PCR product using L1F and L33R for wild-type Col-0 (2502-bp), Bla-6 (4469-bp) and F6 progeny of Rec1 (4469-bp) and Rec2 (2502-bp). **(D)** Assembled candidate region showing the candidate genes At1g65180 and At1g65190 (blue), and the 1967-bp insertion (orange) with its predicted exons (yellow).

Previously, a set of 123 Arabidopsis accessions (five plants per accession) were tested with *On* in order to investigate natural variation in resistance (Gao et al., 2015). From this set of plants, 40 accessions were found to be fully resistant. We randomly selected 19 of these accessions and crossed them with susceptible accessions Col-0 or Sha. The F_1_ plants from 18 of the crosses were susceptible (DI>0). Only the progeny from the Bla-6 x Col-0 cross was found to be completely resistant (DI=0) (Gao et al., 2015; **Figure 1A**).

In the F_2_ progeny (92 plants) of the Bla-6 x Col-0 cross, 72 plants were resistant (R) and 20 plants susceptible (S), fitting a segregation ratio of 3R:1S (**Supplementary Figure 2A**). These F2 plants were genotyped with a set of indel markers (**Supplementary Table 1**). Interval Mapping revealed a QTL at the end of chromosome 1, between markers RH473/474 and RH481/482 (**Supplementary Figure 2B**). Marker RH565/566 was closely linked to the resistance (**Supplementary Figure 2C**). To fine-map the QTL, three rounds of recombinant screening were performed. In the first round, 52 recombinants were identified by genotyping 768 F_2_ plants with markers RH473/474 and RH481/482 (**Figure 1B**i). F_3_ progenies of nine informative recombinants (17 plants per family) were tested in a disease assay and genotyped using 14 additional markers between RH473/474 and RH481/482 (**Supplementary Figure 3A**). As a result, the QTL region was shown to be bordered on the right side by marker SNP51 (**Supplementary Figure 3A**). In the second round, markers RH453/454 and RH565/566 were applied on 528 F_3_ plants derived from the F_2_ plant F-4F (**Supplementary Figure 3B**) heterozygous for the markers in the QTL region, resulting in three informative recombinants (#188, 100 and 102). F4 families of these recombinants were tested with *On* (40 plants per family) and further genotyped with four additional markers within the interval (**Supplementary Figure 3B**). This allowed us to reduce the interval to a 185-kb region between markers RH777/780 and SNP51 (**Figure 1B**ii).

In the third round, 747 plants of the segregating F_4_ family from the heterozygous F3 plant #270 were screened with markers M6 and RH555/556. A total of 15 recombinants were found (**Supplementary Figure 3C**) and further genotyped with markers M14, M24 and M36. Two recombinants (Rec1 and Rec2) between markers M24 and M36 (66-kb region) showed different phenotypic responses to *On*. To confirm these results, we performed a disease assay using the F_5_ progeny (37 plants per genotype) of Rec1 and Rec2 (**Supplementary Figure 3D**). We confirmed a 3R:1S segregation in the progeny of Rec1 and a susceptible phenotype for all progeny of Rec2. PCR products amplified with primers L11, P26, P27 and P33 (**Supplementary Table 1**) from DNA of homozygous F_5_ plants from each recombinant were sequenced. The results showed that the recombination in both Rec1 and Rec 2 had occurred in an interval of 5945-bp between markers M24 and L11 (**Figure 1B**iii). Subsequently, homozygous F_5_ plants fixed for each allele were selfed to produce F_6_ families. We confirmed the expected phenotype of these families in a disease assay using 10 plants per family. By PCR-based sequencing of F_6_ plants, we located the recombination events of these lines at the intergenic region between genes At1g65190 and At1g65200 for Rec1, and 188 bp within the CDS of At1g65180 for Rec2 (**Figure1B**iv).

The region between the two recombination events was subsequently amplified in wild-type (WT) Bla-6 and Col-0, and resistant and susceptible F_5_ progeny of Rec1 and Rec2, using primers L1F (located within At1g65180, **Figure 1B**iv) and L11R (located within At1g65200, **Figure 1B**iv). This yielded a 5,986-bp PCR fragment for Col-0. However, we obtained larger products for Bla-6 and resistant progeny from Rec1, suggesting the presence of an insertion in these plants. With primers L1F and L33R (**Supplementary Table 2**) ~4.5 kb PCR products were obtained in Bla-6 and resistant progeny from Rec1, while the product in Col-0 was 2,502-bp (**Figure 1C**). Using additional primers between L1F and L11R (**Supplementary Table 2**), we sequenced the L1F+L11R PCR product from Bla-6 and identified a 1967 bp-long insertion (**Figure 1D**, **Supplementary Figure 4**) within the intergenic region between At1g65180 and At1g65190. In this insertion, we retrieved (using the FGENESH webtool; Solovyev et al., 2006) a prediction of a potential (partial) gene, although without start codon, with a high similarity to a translation protein SH3-like family protein (AT1G57860). Therefore, two candidate genes are located in the fine-mapped QTL interval responsible for the resistance to *On* in Bla-6: At1g65180, annotated as a Cysteine/Histidine-rich C1 domain family protein, and At1g65190 (Protein kinase superfamily protein *ZRK13*), in addition to the putative gene in the intergenic 1967-bp insertion.

### Targeted mutagenesis of *ZRK13* in Arabidopsis accession Bla-6 results in susceptibility to *On*

**Figure 2:**
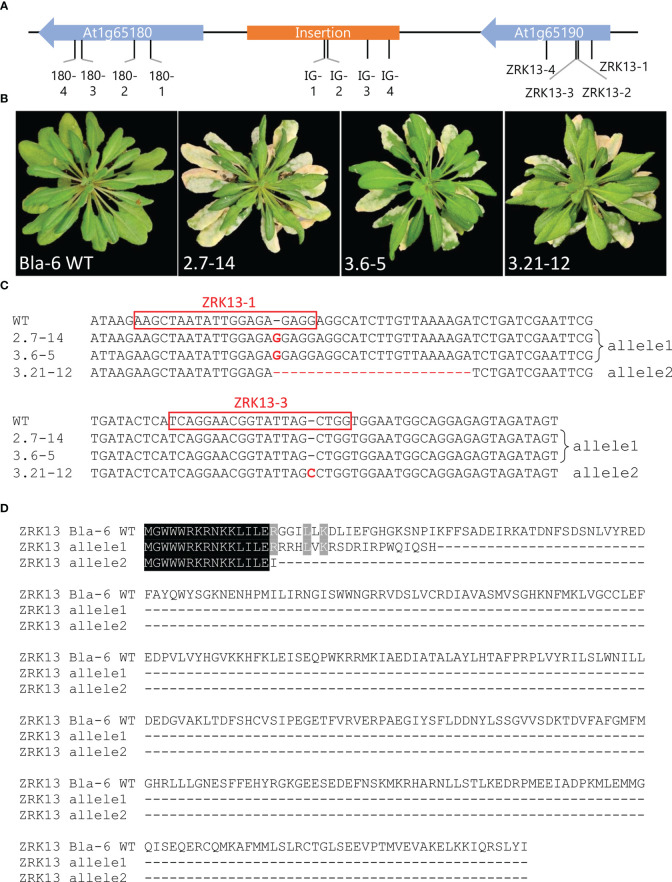
Targeted mutagenesis of *ZRK13* in Bla-6 results in a susceptible phenotype to *Pseudoidium neolycopersici*. **(A)** Location of the single guide RNAs (sgRNAs) in the candidate region of Bla-6. Blue arrows indicate the two genes in the region. Orange square indicates the insertion present in Bla-6. Black bars indicate the location of the sgRNAs. **(B)** Phenotype at 26 days post inoculation of wild-type (WT) Bla-6 and three T2 plants from susceptible *ZRK13* CRISPR transformants. **(C)** Mutation events identified in the T2 plants shown in **(B)** Deletions and insertions in CRISPR mutants are indicated with letters in red. sgRNAs ZRK13-1 and ZRK13-3 are indicated inside red boxes. **(D)** Alignment of predicted proteins of *ZRK13* CRISPR mutant alleles (shown in **C**) compared with wild type (WT) allele of Bla-6.

To elucidate the gene responsible for the resistance in Bla-6, we designed three CRISPR/Cas9 constructs, each containing four sgRNAs targeting one of the candidates (**Figure 2A**). T_1_ CRISPR transformants for each candidate gene were tested in a disease assay with *On*. In total, we inoculated 49 T_1_ plants targeting At1g65180, 53 T_1_ plants targeting the intergenic insertion (IG) and 75 T_1_ plants targeting *ZRK13*. Although most of them would not be homozygous mutant, it was expected that several T_1_ transformants would be bi-allelic mutant. No disease symptoms were visible in any of the T_1_ transformants targeting At1g65180 or IG. However, from the 75 transformed plants targeting *ZRK13*, five plants showed clear disease symptoms (representative transformants 2.26 and 3.21 shown in **Supplementary Figure 5B**). PCR-based sequencing using primers LZRK13F and LZRK13R, amplifying the region containing the target sites of the sgRNAs, revealed the presence of double peaks at the sgRNA ZRK13-1 target site in these five susceptible plants (**Supplementary Figure 5C**). Additionally, we screened at least 15 CRISPR T_1_ transformants of each targeted gene *ZRK13*, At1g65180 or IG to check for mutations at the target sites of sgRNAs. We detected aberrant sequences in two T_1_ plants that showed no disease symptoms; one heterozygous *ZRK13* CRISPR transformant (3.36), and one heterozygous At1g65180 CRISPR T_1_ transformant (2.25) with a mutant allele (**Supplementary Figure 6**).

In a second disease assay with *On* we tested the selfing T_2_ progeny of the five susceptible *ZRK13* CRISPR T_1_ plants (2.7, 2.11, 2.26, 3.6, and 3.21), along with the T_2_ progenies of the resistant heterozygous *ZRK13* CRISPR plant 3.36 (**Table 1**). *ZRK13* T_2_ families 2.14 and 3.37 containing only the wild type (WT) Bla-6 *ZRK13* allele were included as resistant controls. In addition, T_2_ progeny from the At1g65180 CRISPR transformants 2.25 (heterozygous) and 2.11 (WT), plus two T_2_ families from IG CRISPR transformants 2.14 and 2.21, were tested for compromised resistance to *On*. From each T_2_ family, ~40 seedlings were infected with *On.* Susceptible plants were found in all the *ZRK13* T_2_ families, except the controls 2.14 and 3.37 (**Supplementary Figure 5D**). In contrast, no susceptible progeny was observed in the At1g65180 or IG families (**Table 1**, **Supplementary Figure 6C**).

**Table 1:** Phenotype upon inoculation with *Pseudoidium neolycopersici* (*On*) in the T_2_ progeny of the CRISPR/Cas9 transformants.

Gene targeted	Family	# Susceptible Plants	# Resistant Plants
*ZRK13*	2.7	25	14
	2.11	1	39
	2.26	23	17
	3.6	8	31
	3.21	5	30
	3.36	24	16
	2.14 (WT)	0	39
	3.37 (WT)	0	37
At1g65180	2.11 (WT)	0	34
	2.25	0	34
Intergenic insertion (IG)	2.14	0	37
	2.21	0	24

Gene target, family number and number of susceptible and resistant plants per family are shown. WT, wild type.

To pinpoint mutation events in the *ZRK13* gene, we used PCR-based sequencing using primers Det_ZRK13F and Det_ZRK13R (**Supplementary Table 2**). We identified three homozygous mutation events in three different T_2_ families (**Figures 2B, C**). In nine susceptible plants of the T_2_ family 2.7, we identified a 1-bp insertion (G) within sgRNA ZRK13-1 (*ZRK13* CRISPR allele 1). In contrast, PCR-based sequencing of resistant T_2_ plant 2.7-30 yielded a WT allele. In the T_2_ family 3.6 we identified four susceptible plants carrying a 1-bp insertion (G) within sgRNA ZRK13-1, which is identical to the mutant allele in family 2.7. Sequencing of four resistant plants in family 3.6 yielded only WT alleles. Furthermore, in the T_2_ family 3.21 we identified four susceptible plants carrying a homozygous 25-bp deletion, as well as 1-bp insertion (C) within sgRNA ZRK13-3 (*ZRK13* CRISPR allele 2). Sequencing of one T_2_ plant without symptoms (3.21-8) showed it was heterozygous. The predicted protein sequences of the two mutant *ZRK13* alleles indicate identity of only the first 16 or 15 amino acids, respectively, for alleles 1 and 2 compared to the protein sequence of WT Bla-6 (**Figure 2D**). Furthermore, early stop codons are present at amino acid positions 36 and 16, respectively, for alleles 1 and 2.

In T_2_ families 2.11, 2.26 and 3.36 we identified the presence of aberrant sequences in susceptible plants but no homozygous mutation events. In family 2.11, we observed only one plant with disease symptoms after inoculation. PCR-based sequencing of this plant showed the presence of aberrant sequences in sgRNA ZRK13-1. In family 2.26, three susceptible plants showed the presence of aberrant sequences from sgRNA ZRK13-1, while three resistant plants yielded a WT allele. Lastly, in family 3.36, we identified aberrant sequences starting at sgRNA ZRK13-1 in five susceptible plants, while sequencing of one resistant plant yielded a WT allele. We did not observe *On* disease symptoms in any of the 34 tested T_2_ progeny of the *At1g65180* CRISPR transformant 2.25. PCR-based sequencing of five plants from this T_2_ family 2.25 using primers 65180Fw/Rv (**Supplementary Table 2**) yielded only WT alleles, while one T_2_ plant (2.25-18) was heterozygous with one mutant allele containing a 280-bp deletion (**Supplementary Figure 6**).

### Polymorphisms in the *ZRK13* allele of Bla-6

**Figure 3:**
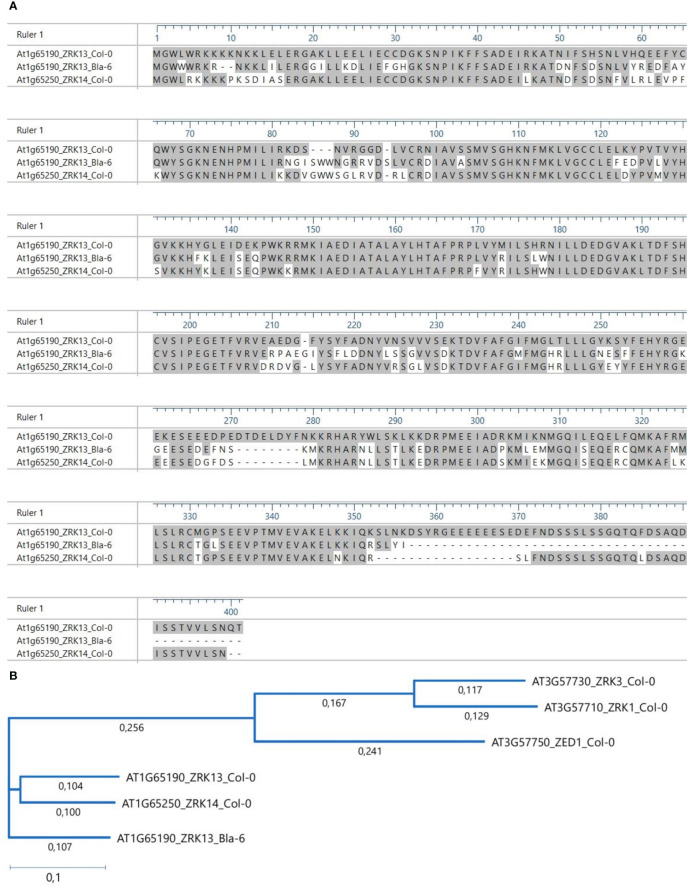
Alignment of the predicted protein sequence of *ZRK13* allele of Bla-6 with the *ZRK13* and *ZRK14* alleles of Col-0. **(A)** Conserved amino acids are shown in grey. **(B)** Phylogenetic tree of some of the *ZRK* genes on chromosome 1 (ZRK13, ZRK14) and 3 (ZRK1, ZRK3, ZED1).

Using NCBI’s ORFfinder the predicted ORF of the Bla-6 allele of *ZRK13* was determined to encode a protein of 346 amino acids (aa), contrasting with the 396 amino acids-long Col-0 protein (**Figure 3**, genomic sequences in **Supplementary Figure 7**). The predicted ZRK13 protein of Bla-6 shows 81.2% similarity with the Col-0 allele of ZRK13 (At1g65190) and, notably, 86.7% similarity with ZRK14 (At1g65250) of Col-0. The relatively low level of similarity of the ZRK13 protein sequence of Bla-6 compared to the Col-0 protein is caused by many amino acid (aa) substitutions and additionally, a 2-aa deletion at position 8, an 8-aa deletion at position 270 and a 4-aa insertion at position 90 (**Figure 3A**). Furthermore, a premature translation termination codon at position 357 causes a lack of the 49-aa disordered region reported to be present in the Col-0 protein according to the domain annotation retrieved from Uniprot. Importantly, when compared with the protein sequences of members of the ZRK family which are characterized to recognize type-3 bacterial-secreted effectors to trigger ETI (ZED1, ZRK1 and ZRK3; Breit-McNally et al., 2022), the ZRK13 allele of Bla-6 clusters in a different clade (**Figure 3B**).

## Discussion

The model species *Arabidopsis thaliana* has been extensively used for screening and characterization of mechanisms of resistance against pathogens. With the aim of uncovering new mechanisms and sources of resistance against *On*, we screened 123 accessions of Arabidopsis for their response against this disease. From the 19 crosses made between resistant accessions and susceptible Col-0 and Sha, the F_1_ of the Bla-6 x Col-0 cross was the only one completely resistant to *On*. To date, the only reported dominant resistance to PMs (*E. cruciferarum*, *G. cichoracearum* and *G. orontii*) in Arabidopsis is conferred by the *RPW8* locus on chromosome 3 (Xiao et al., 2001). In this study we showed that the *ZRK13* gene on chromosome 1 is required for the dominant resistance against *On* in Bla-6.

Through several rounds of recombinant analyses, we fine-mapped the QTL in Bla-6 for *On* resistance and found two candidate genes and an additional potential (partial) gene. We used CRISPR/Cas9 to knock-out the candidate genes, to identify mutations that would lead to compromised resistance in Bla-6. By testing the T_1_ plants transformed with constructs targeting each of the candidate genes in a disease assay with *On*, we identified susceptible phenotypes (*i.e.* showing compromised resistance) only in the progeny of plants transformed with the construct targeting *ZRK13*. Analysis of the T_2_ through PCR-based sequencing allowed us to identify several mutation events. The segregation ratios in disease response in T_2_ families originating from susceptible plants did not correspond to a mendelian segregation. It has been reported that mutagenesis using CRISPR/Cas9 through flower dipping in Arabidopsis may lead to somatic mutations that are not inherited in the sexual offspring (Feng et al., 2014; Jiang et al., 2014). We speculate that some of the mutations in the T_1_ were indeed somatic and therefore were not inherited in the selfing T_2_ progeny. For this reason, it has been suggested that screening of heritable mutations should be done in T_2_ generations or later (Feng et al., 2014). It is important to mention that we were not able to identify homozygous or bi-allelic mutation events in the other two candidate genes (At1g65180 and the intergenic insertion). However, we sequenced only a small number of CRISPR T_1_ transformants. No *On* disease symptoms were found in any of the plants obtained from the transformation using the constructs targeting these loci. Nonetheless, we were able to identify *ZRK13* as essential for the resistance found in Bla-6.

Up until now, four members of the ZED1-RELATED KINASE (ZRK) family in Arabidopsis have been characterized. *ZRK* genes encode receptor-like cytoplasmic kinases (RLCK). Subfamily XII-2 (RLCK XII-2) consists of 13 members (Lewis et al., 2013). Eight of these cluster together on chromosome 3, two are located elsewhere on chromosome 3, and the three remaining members of the family (*ZRK12*, *ZRK13* and *ZRK14*) are located on different positions on chromosome 1. All four characterized *ZRK* members (*ZED1*, *ZRK1*, *ZRK2* and *ZRK3*) are closely related and are part of the chromosome 3 cluster (Lewis et al., 2013). It has been shown that all these four members are required for the activation of ETI through the nucleotide-binding leucine-rich repeat (NLR) protein ZAR1: ZED1 for the recognition of HopZ1a, a *Pseudomonas syringae* type-3 secreted effector (Lewis et al., 2010; Lewis et al., 2013), ZRK1 for recognition of *Xanthomonas campestris* effector AvrAC (Wang et al., 2015), ZRK2 for recognition of *P. syringae* effector HopBA1 (Martel et al., 2020) and ZRK3 for recognition of *P. syringae* effector HopF2 (Seto et al., 2017). Recently, the interaction of RKS1/ZRK1 and ZAR1 has been studied *via* cryo-electron microscopy (Wang et al., 2019), which has allowed the elucidation of the biochemical steps that result in the assembly of the ZAR1 resistosome. This is a pentameric funnel-shaped structure that binds to the plasma membrane eliciting cell death and ultimately resulting in resistance to *X. campestris*. Additionally, it has been reported that ZRKs play a role in the ambient-sensitive immune response in the absence of pathogens. Wang et al. (2017) showed that *zed-1D* mutant displayed a severe phenotype when grown at high temperature by triggering an autoimmune ZAR1-dependent response. Interestingly, in the same study, overexpression of *ZRK13* could partially rescue the *zed1-D* phenotype, strongly suggesting that ZRK13 is able to interact with the ZAR1 resistosome.

The ZAR1 resistosome has been found to have an ancient origin and to be atypically conserved across plants, being found in more than 80 species including monocots, magnoliids and eudicots (Adachi et al., 2020). Therefore, an evolutionary model has been proposed in which ZAR1 remains as a conserved activator of immune responses while RLCKs evolved into a variety of pathogen sensors (Schultink et al., 2019; Adachi et al., 2020; Martel et al., 2020). It remains to be shown whether the interaction of the ZRK13 protein from Bla-6 with the ZAR1 resistosome upon infection with *On* is responsible for the resistance. It would be interesting to study the allelic variation of *ZRK13* across different accessions of Arabidopsis. We have found that the predicted protein of *ZRK13* in Bla-6 holds many polymorphisms when compared to Col-0. However, confirmation of the allelic variant in Bla-6 at the mRNA level will help to confirm this information.

Although the Arabidopsis-*On* interaction is less well-characterized compared to the other PM pathogens infecting Arabidopsis, our study shows that it represents a robust model that allows analysis of new mechanisms of resistance against biotrophic pathogens. The identification of *ZRK13* as the gene required for resistance against *On* in Bla-6 opens new opportunities to elucidate the molecular mechanisms of this interaction. Most importantly, the interaction of ZRK13 with ZAR1 can be tested by generating *zar1* mutants in a Bla-6 background and test their response against *On*. Additionally, overexpression of the ZRK13 allele of Bla-6 can be tested in a Col-0 background to confirm its function in resistance.

## Data availability statement

Sequence data generated in this study can be found in the GenBank repository with accession number OP897811.

## Author contributions

Conceived and designed the experiments: YB, RH, A-MW, MSM. Performed the experiments: MSM, RH, DG, MA, ID, GS, XW. Data analysis: MSM, A-MW, YB. Writing of the paper: MSM. Critical review of the paper: YB, A-MW, RV. All authors contributed to the article and approved the submitted version.
