# Supplementary material for: ZED1-related kinase 13 is required for resistance against Pseudoidium neolycopersici in Arabidopsis accession Bla-6
Source: Front Plant Sci. 2023 Mar 21;14:1111322. doi: 10.3389/fpls.2023.1111322 (PMC10071312; doi:10.3389/fpls.2023.1111322)
Supplement: Supplementary file 1 [file DataSheet_1.docx]

Supplementary Material

# Supplementary Tables

Supplementary Table 1. Primers used for QTL analysis and mapping of the candidate region on chromosome 1 of Bla-6. Name, location in the reference Col-0 genome and sequence are provided.

**Supplementary Table 2**. Primers used for sequencing of the candidate region on chromosome 1 of Bla-6. Name, location in the reference Col-0 genome and sequence are provided.

**Supplementary Table 3**. Single guide RNAs used for CRISPR constructs. Name, sequence and target gene/locus are indicated.

**Supplementary Table 4**. Plasmids used for CRISPR/Cas9 construct. The name of the plasmid, description and sources are indicated.

## Supplementary Figures

**Supplementary Figure 1.** Disease severity index (DSI) scale of powdery mildew *Pseudoidium neolycopersici* on *Arabidopsis thaliana*.

**Supplementary Figure 2.** Analysis of Bla-6 x Col-0 F2 progeny. **A**. Segregation of resistant (DSI = 0) and susceptible (DSI > 0.5) F2 plants. **B**. Preliminary QTL analysis of the F2 of the Bla-6 x Col-0 cross. LOD score and position in centimorgans (cM) are indicated. **C.** Average disease index (DI) score on the F2 per genotype class for marker RH565/566. B, homozygous Bla-6; H, heterozygous; C, homozygous Col-0.

**Supplementary Figure 3.** Informative recombinants in each of the three screenings carried out to map the resistance locus in chromosome 1. **A**. Recombinant screening 1 of Bla-6 x Col-0 F_2_ population. **B**. Recombinant screening 2, performed using the F_3_ progeny of recombinant F-4F. **C**. Recombinant screening 3, performed using the F_4_ progeny of recombinant F-4F-270. B, homozygous Bla-6 allele, C, homozygous Col-0 allele, H, heterozygous, R, resistant, S, susceptible, seg., segregating for resistance against *Pseudoidium neolycopersici* (*On*). **D**. *On*-infected plants of Bla-6, Col-0, and F_5_ progeny of recombinants Rec1 and Rec2.

**Supplementary Figure 4.** Sequence of the Bla-6 candidate region including the intergenic insertion. Genes At1g65180 and At1g65190 are highlighted in green; the intergenic insertion in Bla-6 compared to Col-0 is highlighted in yellow; region showing homology to an SH3-like gene is underlined; sgRNA target sites are indicated in bold; primers flanking the ZRK13 sgRNA targets are shown in bold italics.

**Supplementary Figure 5.** Targeted mutagenesis of *ZRK13* in Bla-6 results in a susceptible phenotype in the T_1_ and T_2_. **A**. Location of the single guide RNAs (sgRNAs) in the *ZRK13* gene (At1g65190) of Bla-6. Blue arrow indicates the *ZRK13* gene. Black lines indicate the locations of the sgRNAs. **B**. Phenotype of wild-type (WT) Bla-6 and two susceptible CRISPR:ZRK13 T_1_ transformants at 12 days post-inoculation (dpi) with *Pseudoidium neolycopersici* (*On*). **C**. Sequence trace files of the plants in B showing the sgRNA ZRK13-1 (red box) region with double peaks present within the targeted region. **D**. Trays of T_2_ families 2.7 (carrying *ZRK13* mutant allele 1) and 3.37 (carrying wild-type *ZRK13* allele) at 27 dpi with *On*.

**Supplementary Figure 6.** Mutant allele of At1g65180 in heterozygous CRSIPR transformant 2.25. **A**. Graphical representation of gene At1g65180. Orange arrow, coding sequence; blue arrowheads, sgRNAs; red line, sequence deleted in mutant allele in T_2_ CRISPR plant 2.25-18. **B**. Sequence trace files of T_2_ progeny homozygous for the wild-type allele (2.25-9) or heterozygous (2.25-18). **C**. Tray with *Pseudoidium neolycopersici*-inoculated plants of T_2_ family 2.25, 27 days post inoculation.

**Supplementary Figure 7.** Pairwise alignment of the genomic sequence of *ZRK13* of Col-0 and Bla-6. Primers for sequencing are indicated in blue rectangles, start codon in green rectangle, and stop codons in red rectangles.
