# Supplementary material for: ZED1-related kinase 13 is required for resistance against Pseudoidium neolycopersici in Arabidopsis accession Bla-6
Source: Front Plant Sci. 2023 Mar 21;14:1111322. doi: 10.3389/fpls.2023.1111322 (PMC10071312; doi:10.3389/fpls.2023.1111322)
Supplement: Supplementary file 2 [file Table_1.docx]

**Table S1. Primers used for QTL analysis and mapping of the candidate region on chromosome 1 of Bla-6**.

Name, location in the reference Col-0 genome and sequence are provided.

| Name | Type | Location (TAIR10) | Forward primer | Reverse primer | Restriction enzyme |
| --- | --- | --- | --- | --- | --- |
| RH555/556 | Indel | 3212189-3212389 | GGCTTTCTCGAAATCTGTCC | TTACTTTTTGCCTCTTGTCATTG |  |
| RH473/474 | Indel | 24329048-24329148 | TTACCAAAAGGTTGCGAACA | TGTTTTGTCTTTATCAACTCCACAA |  |
| RH565/566 | Indel | 24370345-24370540 | CTGCCTGAAATTGTCGAAAC | GGCATCACAGTTCTGATTCC |  |
| RH481/482 | Indel | 25154371-25154490 | GCCCAGAGAACTAAGTCAGCA | GAAGCCAGAGAAAAGGCAAG |  |
| RH569/570 | Indel | 29011992-29012148 | GCATCGCTCTTAAACAACCAT | CGTTGCAAAACCGTATCAGAA |  |
| RH777/780 | SNP | 24171640-24172127 | CGACAAAAGTGAAGAAATAAATGTG | CGCAAAAACGAATGATGGTT |  |
| M24 | CAPS | 24213389-24213762 | CCCTTCCCACAAGTAAAGCA | AATCGGTGGGGTAAATGTTG | *MspI* |
| M36 | CAPS | 24278646-24279530 | GCATTTGCACAAATTAAGAAAAA | GCCACCATGGGAATAGGTT | *HinfI* |
| SNP51 | SNP | 24357325-24357444 | GTCTAAACCGCACTCGCAAT | CTCGCACTTTGCCACTATCA |  |
| M6 | CAPS | 23981483-23981846 | CCCCTGACACCACAAGAGAT | TGGATGAGTTGTATGATTTGAGAGA | *RsaI* |
| M14 | CAPS | 24029663-24030011 | CGTTGAGGGTAGGACACAGG | AAATTCTCACTCCCACACACG | AluI/*DdeI* |
| Bla-6-1 | Indel | 24347660-24347770 | CCAAGAAATTGAACGCAACA | GCCGATTTTTGTTTTCTTAGCA |  |
| Bla-SNP42 | SNP | 24364379-24364533 | TGTCAGCCATTGGAGACAAG | CTTATTGCAGGTCCCGATTG |  |
| Bla-SNP45 | SNP | 24385803-24385973 | AAAAATGCGCCAATTCAAAG | GGAAATAGCAAAAAGCGAAAA |  |
| Bla-SNP17 | SNP | 24430385-24430507 | TATGGACCCTGGAGCAAATC | ATTTCTTGCATGGGGATGAA |  |
| Bla-6-4 | Indel | 24507047- 24507181 | GTGAGGGAATAGGGACATGC | GCCAGCTTGCAATTCATTTA |  |
| Bla-SNP30 | SNP | 24576433-24576627 | TTCAACCCCACTTTTGATGA | GCCCGACTCGATGAATAGAA |  |
| Bla-SNP14 | SNP | 24615974-24616076 | GGCTCTTCCCTCCTCTGTCT | ATGAACGATTGCGGTTGTG |  |
| Bla-SNP19 | SNP | 24666538-24666702 | CCTGAACCTGGTGCTGCTAT | CGACCAAGGTCGCTAGATTT |  |
| Bla-SNP12 | SNP | 24710590-24710736 | TCATCCAAATGGTGTTTTCG | AGCTTAGTCACCCGGGATCT |  |
| Bla-SNP21 | SNP | 24764515-24764661 | TCACTGGGCTGTCTTCTGGT | TACCAACCAAGACGGCTTTT |  |
| Bla-SNP9 | SNP | 24808129-24808249 | GGAGCGGTCAAGACCATATT | TTGCATTTGCTTTTTGCATC |  |
| Bla-SNP22 | SNP | 24854680-24854876 | TCCCAACCATGTTTAGGTAGC | TGCCTTCAATTCACTCACTCC |  |
| L11 | Sequencing | 24218618-24219333 | TCAGCTCAAGAAAGACGAAGG | TGCAGATTCAATCAGAGACCTG |  |
| P26 | Sequencing | 24234230-24235115 | TCAGCAGACCCAAATTTCCTA | TGAGACTAGGCCACTCCTAACG |  |
| P27 | Sequencing | 24243231-24244005 | GGTGCTAACATCTTGGCAGTT | CTACCTTGGCGCCTGCTC |  |
| P33 | Sequencing | 24262525-24262858 | CGTCGATTCTCCGATAGCTC | CCACAAAGCTCTGATCAAAGAAG |  |
| L1 | Sequencing | 24213348-24213897 | CCAAATCGCATTCATGGCAT | TCTCCGTCACATGAGCTAAGAG |  |
