# Supplementary material for: ZED1-related kinase 13 is required for resistance against Pseudoidium neolycopersici in Arabidopsis accession Bla-6
Source: Front Plant Sci. 2023 Mar 21;14:1111322. doi: 10.3389/fpls.2023.1111322 (PMC10071312; doi:10.3389/fpls.2023.1111322)
Supplement: Supplementary file 3 [file Table_2.docx]

**Table S2. Primers used for sequencing of the candidate region on chromosome 1 of Bla-6**.

Name, location in the reference Col-0 genome and sequence are provided.

| Name | Location TAIR10 | Primer sequence |
| --- | --- | --- |
| 65180Rv | 24211765 | CAAACACACTTACAAATCTAAATCACA |
| 65180Fw | 24213874 | TCCGTCACATGAGCTAAGAGAA |
| L21 Fw | 24213536 | TTCACAGCGCTGACATTCAT |
| L22 Fw | 24214060 | CAACATCAACGAAGCACGAT |
| L23 Fw | 24214533 | CCAAATTATGCAACGGATGA |
| L24 Rv | 24214862 | TGTTGACTTGAAGAAATAAAGTTTTGA |
| L25 Rv | 24215376 | TCTCCGATCCAAAACCCATA |
| Det_ZRK13F | 24215449 | GGTGTTGTGCGTCACCTAGA |
| LZRK13Fw | 24215788 | GGAGGACGTCAATGTTGAAAA |
| L26 Rv | 24215726 | TGTGAATCTTGTATTGATGATTGTCT |
| L27 Rv | 24216285 | GGGTCAAATCTTAGAACAAGAGC |
| L28 Rv | 24218678 | AACCGGTGGATCAGTCTCAG |
| L31 Rv | 24214344 | GGGTAACATGATTTAGTTATGAGGGTA |
| L32 Rv | 24215306 | TGTCCAAAATAAAGAATGATGTTCA |
| L33 Rv | 24215849 | AGTGCTACGTTACTTTTGATTCATT |
| L34 Rv | 24216115 | CGACTCTTCTTCTCTTTCTTCAGG |
| L35 Fw | 24214616 | TTCTTGGCACTTCCTTAATCG |
| Det_ZRK13R | 24217397 | TGAATTTTCGCGGGAGGA |
| LZRK13Rv | 24217402 | CCCAAACACACATACACAAACTC |
| L36 Fw | 24217571 | TTCAAACACTCGGCTGGAAT |
| L37 Fw | 24218024 | CTTTGATTGGAAGTCTCCCTCT |
