# Supplementary material for: ZED1-related kinase 13 is required for resistance against Pseudoidium neolycopersici in Arabidopsis accession Bla-6
Source: Front Plant Sci. 2023 Mar 21;14:1111322. doi: 10.3389/fpls.2023.1111322 (PMC10071312; doi:10.3389/fpls.2023.1111322)
Supplement: Supplementary file 4 [file Table_3.docx]

**Table S3. Single guide RNAs used for CRISPR constructs.**

Name, sequence and target gene/locus are indicated.

| Name | Sequence | Target |
| --- | --- | --- |
| 180-1 | TTCCCATCAGTATAAGCCGG | At1g65180 |
| 180-2 | ATGGGCTTCGATCTCCATGG |  |
| 180-3 | TTTGGTGATGTGTAAAACAA |  |
| 180-4 | TAGAGCGCATTTGAAATCCA |  |
| IG-1 | TCAGGAAGAAGCAGAACGAG | Intergenic Insertion |
| IG-2 | GAGCATGTGCAACAGTCTCA |  |
| IG-3 | GCAATTGTGTGAAGCTGCAA |  |
| IG-4 | GAAACGAACAAAGAAACCTG |  |
| ZRK13-1 | AAGCTAATATTGGAGAGAGG | At1g65190 (*ZRK13*) |
| ZRK13-2 | CCGTTCCTGATGAGTATCAT |  |
| ZRK13-3 | TCAGGAACGGTATTAGCTGG |  |
| ZRK13-4 | CCTCTGCTGGTCTCTCAACC |  |
