# Supplementary material for: ZED1-related kinase 13 is required for resistance against Pseudoidium neolycopersici in Arabidopsis accession Bla-6
Source: Front Plant Sci. 2023 Mar 21;14:1111322. doi: 10.3389/fpls.2023.1111322 (PMC10071312; doi:10.3389/fpls.2023.1111322)
Supplement: Supplementary file 5 [file Table_4.docx]

**Table S4. Plasmids used for CRISPR/Cas9 construct**.

The name of the plasmid, description and sources are indicated.

| **Plasmid** | **Description** | **Source** |
| --- | --- | --- |
| pICSL12015 | AtUBI10 Promotor for Cas9 | Jonathan D. Jones (Castel et al., 2019) |
| pICSL60004 | rbcS-E9 Terminator for Cas9 | Jonathan D. Jones (Castel et al., 2019) |
| pICSL70008 | FastRed (FastR) Construct | Nicola Patron (Engler and Marillonnet, 2014) |
| pICSL11024 | NPTII | Jonathan D. Jones |
| pICH47742 | Backbone for Cas9 | Sylvestre Marillonnet (Weber et al., 2011) |
| pICH47751 | Backbone for FastR | Sylvestre Marillonnet (Weber et al., 2011) |
| pICH47761 | Backbone for sgRNA1 | Sylvestre Marillonnet (Weber et al., 2011) |
| pICH47772 | Backbone for sgRNA2 | Sylvestre Marillonnet (Weber et al., 2011) |
| pICH47781 | Backbone for sgRNA3 | Sylvestre Marillonnet (Weber et al., 2011) |
| piCH47791 | Backbone for sgRNA4 | Sylvestre Marillonnet (Weber et al., 2011) |
| pCRISPR-Pink | pAtU26-6-CRISPR pink module | Mark Youles (TSL Norwich, Sybio) |
