# Supplementary figures and images for: ZED1-related kinase 13 is required for resistance against Pseudoidium neolycopersici in Arabidopsis accession Bla-6
Source: Front Plant Sci. 2023 Mar 21;14:1111322. doi: 10.3389/fpls.2023.1111322 (PMC10071312; doi:10.3389/fpls.2023.1111322)

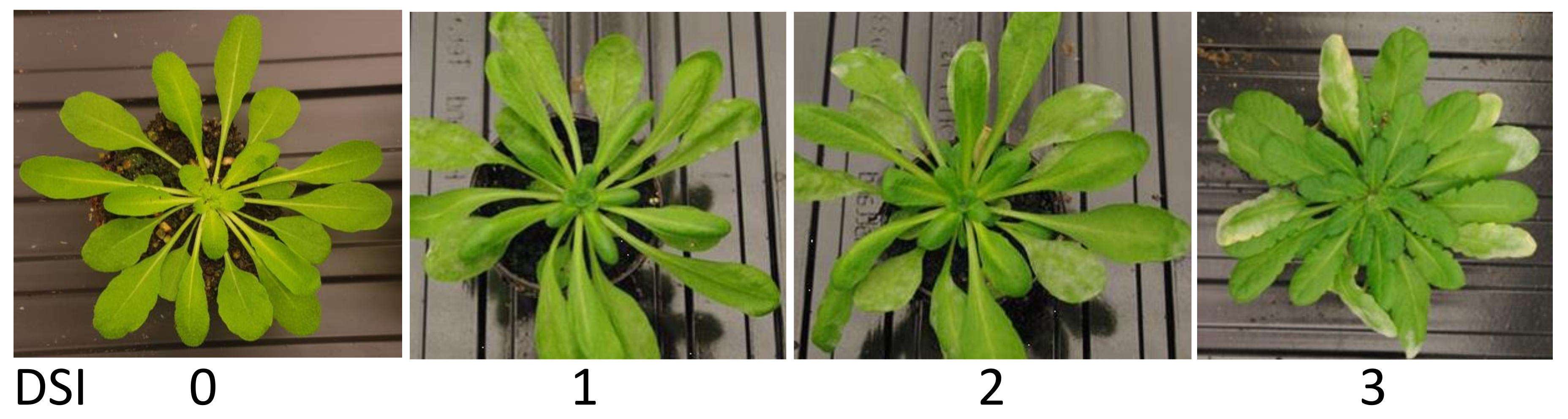

Supplement: Supplementary file 6 [file Image_1.tif]

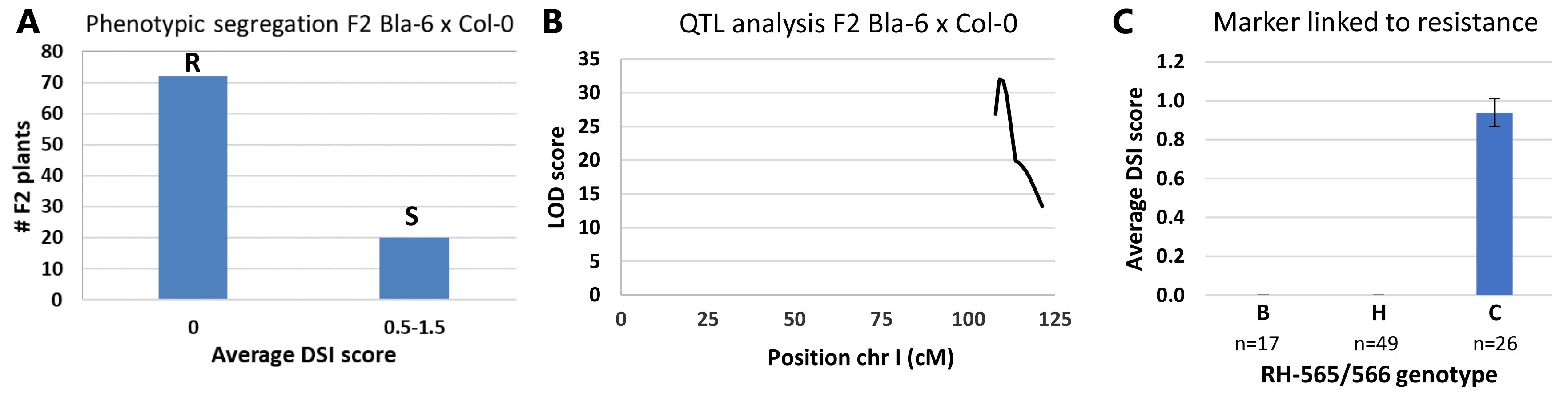

Supplement: Supplementary file 7 [file Image_2.tif]

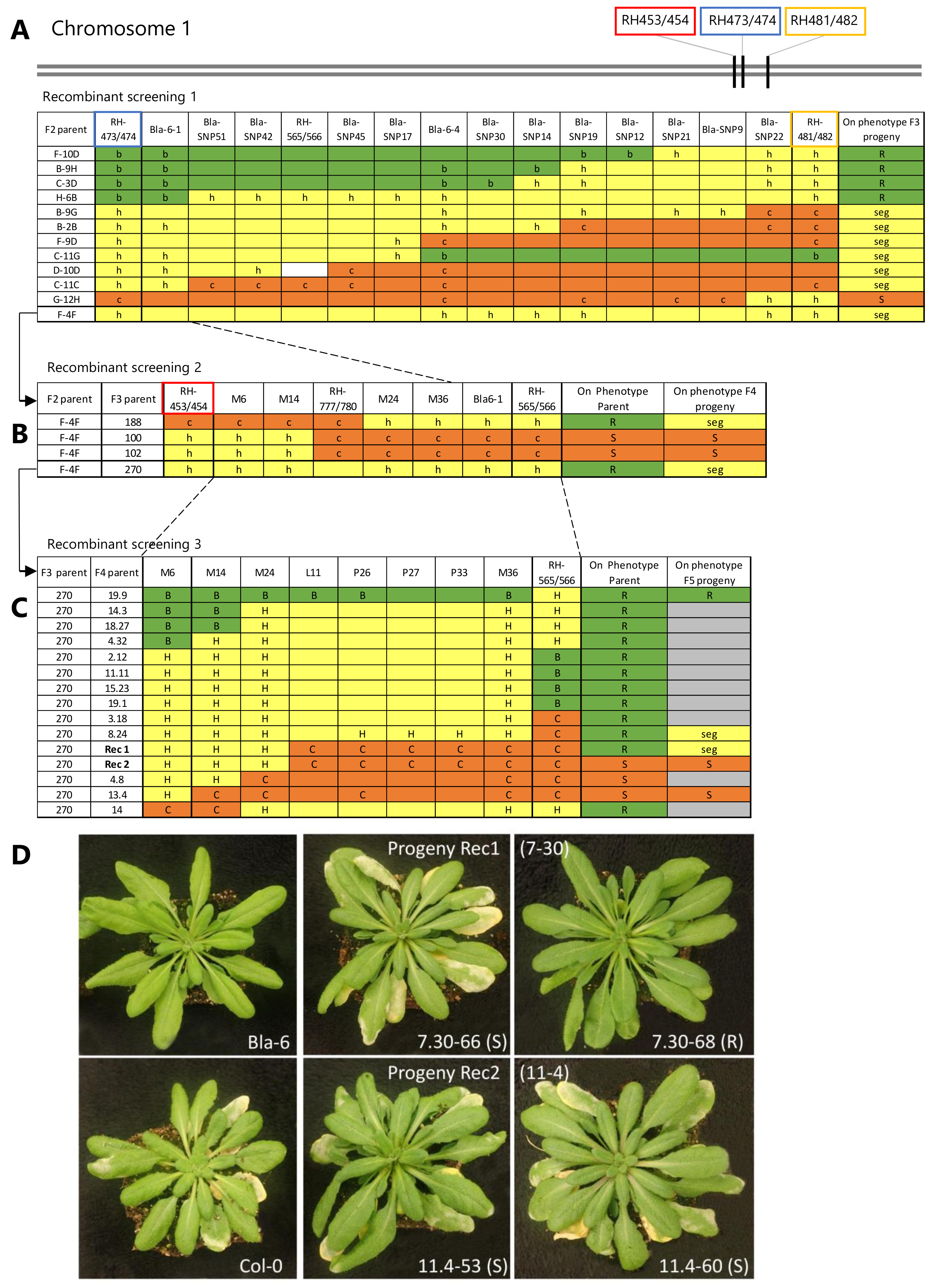

Supplement: Supplementary file 8 [file Image_3.tif]

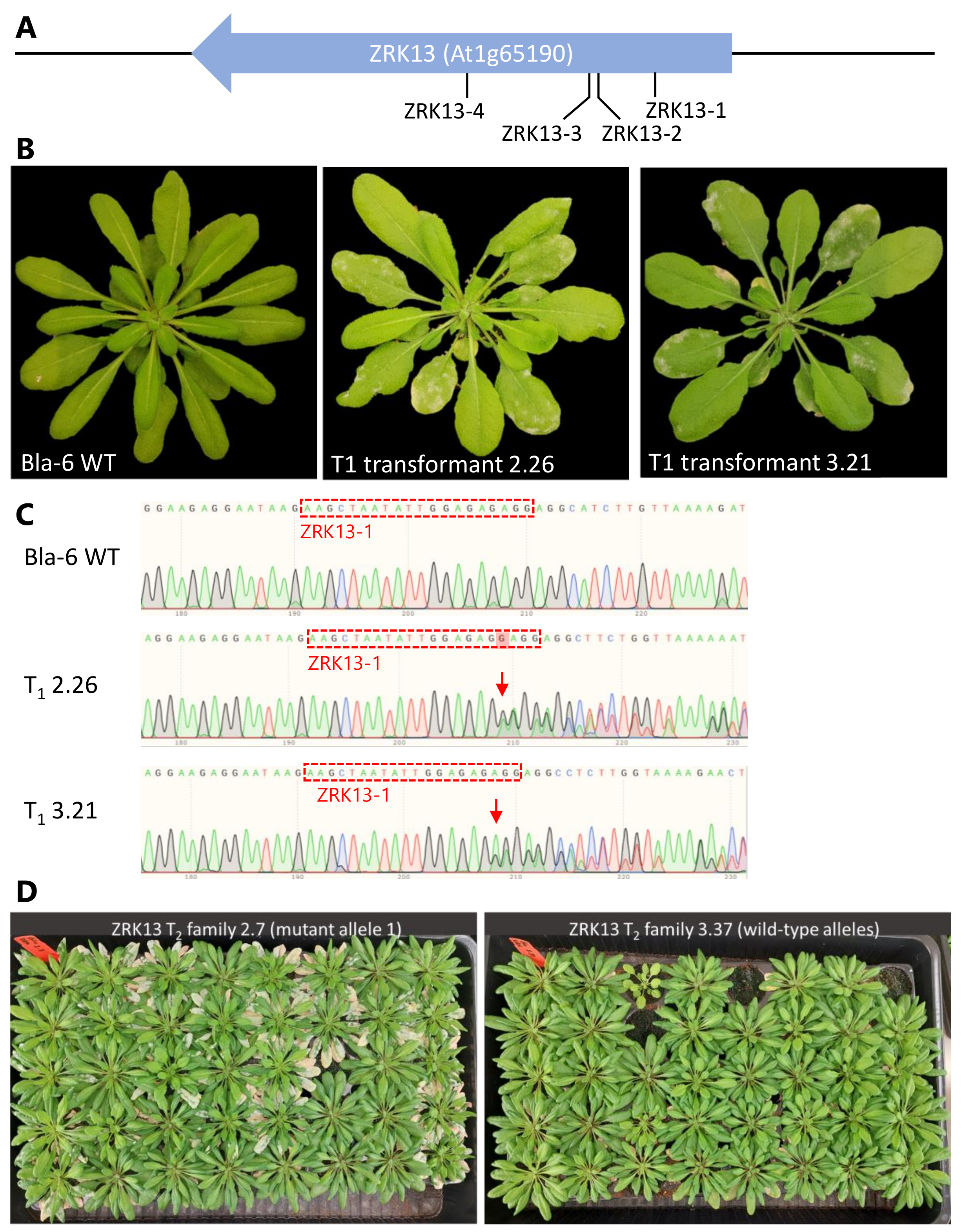

Supplement: Supplementary file 10 [file Image_5.tif]

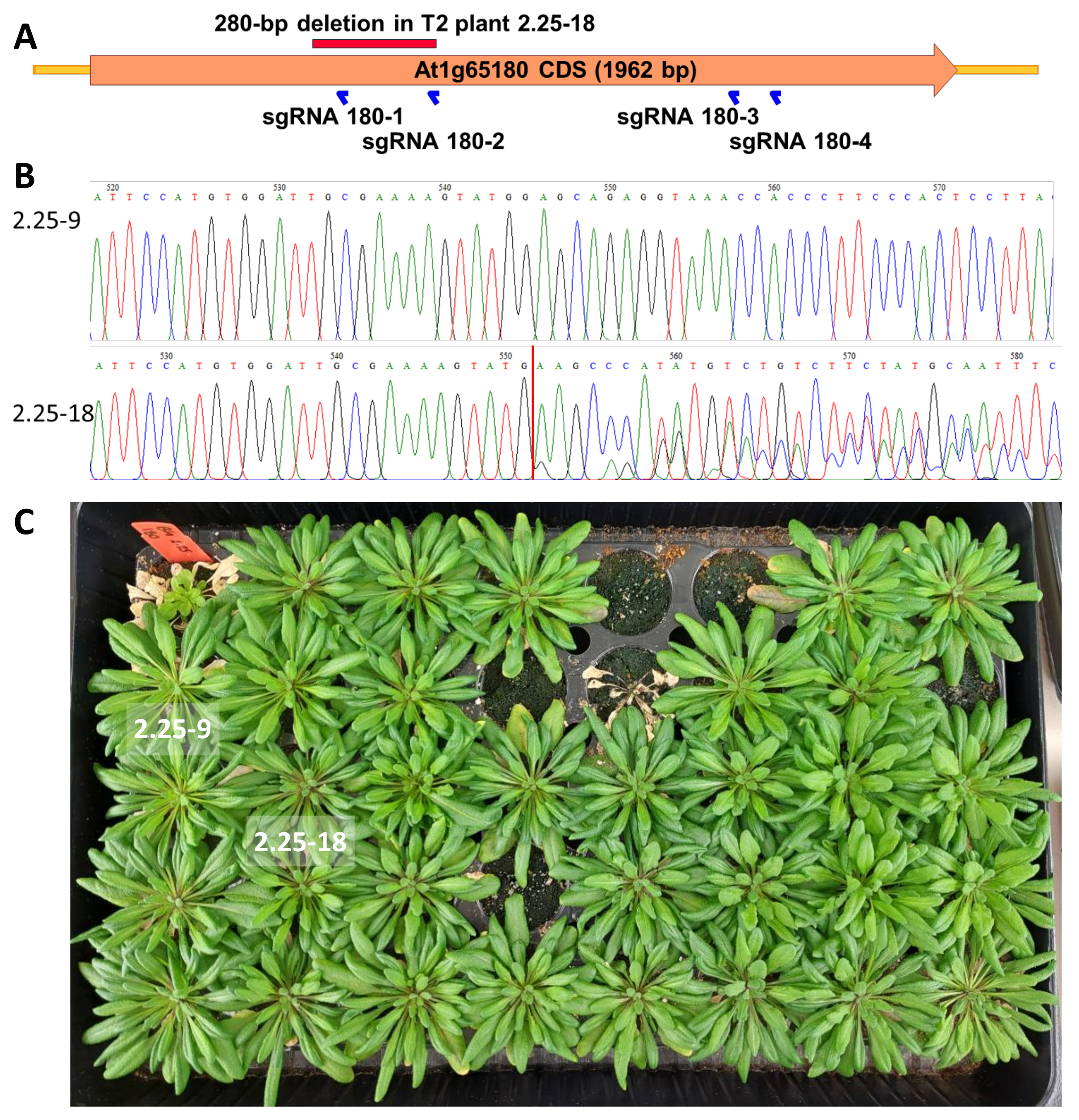

Supplement: Supplementary file 11 [file Image_6.tif]
