# Supplementary material for: ZED1-related kinase 13 is required for resistance against Pseudoidium neolycopersici in Arabidopsis accession Bla-6
Source: Front Plant Sci. 2023 Mar 21;14:1111322. doi: 10.3389/fpls.2023.1111322 (PMC10071312; doi:10.3389/fpls.2023.1111322)
Supplement: Supplementary file 9 [file Image_4.pdf]

Supplementary Figure 4. Sequence of the Bla-6 candidate region including the intergenic insertion. Genes At1g65180 and At1g65190 are highlighted in green; the intergenic insertion in Bla-6 compared to Col-0 is highlighted in yellow; region showing homology to an SH3-like gene is underlined; sgRNA target sites are indicated in bold; primers flanking the ZRK13 sgRNA targets are shown in bold italics.

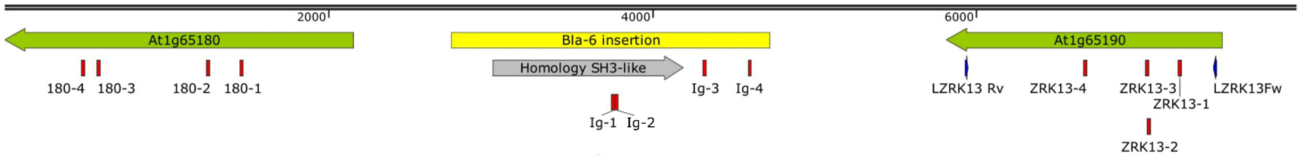


```
ATGTAGCGCGGATCCGATCCAAGTATACGTAAAAAAATTACAAACGTGGAAACAAAAACAACACCAAGATCAATTTTAGT   80
TGTAGAAGGGAAGGGTCTTAATGTCACAACAACTAATAGAGCAAAAGTATTTATCTTCGTCGGTGACTTTCAAGATGATA  160
GGGAAAATGCAATGGTACTTACATTTATAACAAAGTGGTCGAGACATACTATTGTTGCGAACCGTCTCGTAAAGTGTCAT  240
CTGCATCCGGTCATCATCCAATACTATATTTGTTTCCGGTTCCAGACCTCGAAAATCTCCGAGCACACAATTGGTATGCA  320
AAGTTACCCCACAATCTTTACAAGTGTAAAACCATTTTGTTGGATCAGTTTCTTTCTCGCAAATATCACACCAATATTTA  400
CCACTTGCATCTCTTTCACCAAAGCATAAAGAGAGAAAATGATCCTCGACTCTATGCTTGACCTCATGTGGTAATAGAGC  480
GCATTTGAAATCCAAGGCGTATCCACACTTATCTTCAACGCATCTGAGCACGTGAGATGTTTCCTTGTTGCAAGCACTAC  560
ATACTCCTTTTGGTGATGTGTAAAACAAGGGATGTTGGTGACTTGGATGGACAAGTGACCCAGAAAAGGAGGCACATCGC  640
ACATCTAATCCAAACGGAAAAGTACCGTATATGAAACAATTAAAAAATACTCCACAAGCCTTACACTTAGAGTAGTTACG  720
TTGAGATGCATGTAGAATATATGGTTTATTGCTTAGCACATGACGTTTTCGTGTAGGATGTTTAGCACAGTTCTCGTGTA  800
GAAGAAAATCACAACTCATACAACTGTAGAAGGGATCATGATAGATAGGATAAGCACATGCCTTGCAGCTTCTCTCCTCA  880
ACAAAGGTACCAGACTTGTCGAGTTTTAAGTTATGCTCTGTATGGCTGAAATGTCGTATTATATCTTCATCAATCACCTC  960
GAATGGTTCCATAGGTTCATCTTCTTCAGGCTCATCTTTCATCTCTATTCCATCCCAAACATCTTCTCTAGTAGCGCATT 1040
TGGTATGAAACGTAGACTCAGAACACCTCTTACAAGAATAGCCCCCACAACTCCAATCCATTTTCTTACGACAAACTCCG 1120
CACACTGAATCAATAATACCAAGAAGAGAAGTGCGAGAAACGCGATGATCATGGCGGTTGATATTGATGACCCATGGAAA 1200
TCCAGAGCAATTGTTGTGGGAAGTGAAATTGCATTGAAGACAGACATATGGGCTTCGATCTCCATGGAGGCCGCAAGTGG 1280
TGCAAGTAAAAGAGATCAATTTTGGCATAAGGGTTAGTTCATGGTTGTGGGTGTTTTGGTCGTGAAGATTTAGTGGTGGT 1360
GGAAGAGAAACACAGCGCAAATCCAAAGTAAAGTTACAAGCGGAACAGTGATAAAACATTTCGTCAAAAATCTTTTTCCC 1440
ACATAAACTGCAATTCCCATCAGTATAAGCCGGTGGTTTACCAGTGATGAGTTTTAGTGTGTGTAAGGAGTGGGAAGGGT 1520
GGTTTACCTCTGCTCCATACTTTTCGCAATCCACATGGAATGCCAAATCGCATTCATGGCATTTGTAAGGATATCCACTG 1600
CCTCCCTTCCCACAAGTAAAGCAGGTGAAGGAGCTTCTCACCATCTCTAGCTTGAGTTTGTGGTCATGGTTCTGGGGGAA 1680
ATCAATGACCTCCGGTGGTGGGTATTTGACACAATCCAAATGGATGTTGAATTCACAGCGCTGACATTCATAGTTCTGGA 1760
TATAGGGCTGGATTGTCTCTTTACATGTACGGCATCTCACACTCCAGCTGGCAAAATTTGTAATAAGAGAAAGATTCTTA 1840
TCACACTTGTGAGAAGGGTGGTTGATCTCTTTAGGGCAGTTGCTGCAACTCTTATGGAAGAAATAACGACATTTTCTGCA 1920
GTTGTAGCCATCACTGATAGTTTTGAATTGGAGTCCACAACATTTACCCCACCGATTATCTCTTGCAGGTGTCAGCCAGT 2000
GCTCGTGAATGGGTAGTCGCACCATCTCAGGCTCCATGGTTACTAAGAGATCTAGTGAGAATTTCTCGTTGAACTTTTGG 2080
TCTCTGATTTCTCTTAGCTCATGTGACGGAGATAAGTGAAGAAAACAATACTAGTTATTTTATTTAGTACTCATTTAATG 2160
GTTAATTATATGTTTTTTTATTATATATTAGTAAAAGAAAGTTTAGATCTGACATGGTAATCACTAAACTATTCATCATTTG 2240
ATTTTGATAACGTGCCAACATCAACGAAGCACGATTTACATTGCAAAAGACTTTCTATTTAATAGTAGAGTTTTAATGGG 2320
TCAATAAATAGTTGTTACATTATTGCAACAAAACAATAATAAATATTAAATCTGACAGATAACTGCTACCTACTCAACAT 2400
TATATCGAACACGATAAATCGATAAATCAATAGAAGCACAATCGATGCAATCAAGTTTTTTCTTAATAGCTTTCTAAATA 2480
CAAGATGATTTGTGATTTATAATTTGTTAAAATAAAGACAGATATTACTAAAACCTACCCTCATAACTAAATCATGTTAC 2560
CCTCTCATAAAATAATGAACGTTGGGTCATCTAAGAAAAACTCTCAGTGCATAATAATGCAAGTTACTTGGATCCCATGA 2640
CCATTGAAAGTACAGAGAACAAAGATACATGAATACATGATACTTATATGAATCAATAAGAAAGAAACTTTAAATATTAG 2720
GATACCAATCTCCATATTATGCCAAGGATGAACAGTAGCTATAATTAGTCTGTTCGCTTCGTCGCCGCAGGTAGTTGCGG 2800
CTGCGGCAGACACAGATGAAGCTGTTCGTTTCACTACCGCAGATAGATGCGGCAAAACGCTGCGGCCGACGCAACGTTAT 2880
GCGGCGAAAAAAAATAAACGCCGATTAAATTCAGCGTTAGAATTAAAAAGCTGCGATTAATATGCCAAATGACAGAAATA 2960
CCCTTAATATATTTAGAAAACAACGATAAAACCTAACTCTAAAACCTAACACATTCACGATCTGAAACTTTTCTTCTCTT 3040
TTACACGATTTCGATCTGAAAAGTAAAGCTCAGCGATTTCGAATTAAACTCGGCGATTTCGAAGTAATGGGTTTTGTTTT 3120
AGAAGTTCTCTGGTTGACCCTGAAGAACAAGAGATCTGTTCGCGAGACCATTCAGGAAGAAGGGTTATATTCTACTCTCC 3200
ACTTACCTCATAACCTTCAAGGTCGGTGATTACGTCAATGTCAAGGTTAATGGAGCTATTCACAAGGGTATTCCTCATAA 3280
GTTCTACCATAGTCGTATTGGTCGTATCTGGAATCTCACTAAGCGTGCCGTTGGTGTTGAAGTCAACAAACAGGTTTCGA 3360
TTACTCAAAATTCTCATCTGTTTGGAAGAGTTTTGATTGTTGAAATTAGGATTTGAACGTTATTTTGATTCTTTGATTGG 3440
GGTTTAATTCGTCTTTGTGTGAGCTATCTGTTTTGAATTTTGTGATGTGTGATTAGTATTGGTTTGAAAGCTTCAAGCTT 3520
TTCTTCTTGGGTCCTTCTTTTGCTACTTGTATAATTTGCAATCAATCTACTATGATTCTTAGTGATGTTTTCATTTTGAC 3600
```

TTTGATGTTTATCATTTAGTTGAATCTCTTGGGGAGCTAATTTTGAGTTTTGGGTTGGGTTTTAGATTAAGAACAGAATC 3680
ATAAGGAAGAGGATACATGTACGTGTGGAGCATGTGCAACAGTCAAGGTGTGATGAGGAGTTTAATC**TCAGGAAGAAGCA** 3760
GAACGAGTG**GAGCATGTGCAACAGTCTCA**AGGTGTGATGATGAGTTTAGTCTCAGGAAAAAGCAAAACGATGTGTTTAAG 3840
GCTGCTGCTAAAGCTAGATGTGAAACTATTAGCACTAAGAGACAACCTAAAGGTCCTAAACCTTGTTTCATGGTTGAAGG 3920
TATGACCTTGGAGACTGTCACTCCCATTCCCAATGTTGTCAACGATCTCAAAGGTGGCTATTGAGTTGTTTCCATTTTTT 4000
TCATTCTCTTCTAAGAATTTTTGCACTTGCTTTGTTCTTTTTATGATGTTTTTGTATCAAACAAGACTGAATTCTATATT 4080
GTACGAGTTCAGATTTTGTTAGGAAAGATGGTTTATCCCAATCCTTTACCTGTTCCATTTGTATTTAAAATCGTTTCTGC 4160
ATTTTTCAGAAAACTGATACTTTGGGAAAAGTTGAGACATCTAATCTCTTTCAGTGTTAGCAATTTATACCACACAAAAA 4240
TAAACAGAGAGAGCAATCGAAACAAAATCTCTTTGTAAAGTTGGGAAGTGCAAACAGACCTTTTAATACCT**TTGCAGCTT** 4320
CACACAATTGCCTCCTCTTCATTCATTAGTTAAATGTTTGAGTTTGCTATTTTTGTTTTAACTAAGCTTATGATTTATCA 4400
CAAAATTTTATTGTTATAATTATATATATTTAACTAAAAATTATTACTCCTTACCAAAAAAAAACTAAAAACTATTACTA 4480
GCTAAACAAATATTTTTTATGGATGTAAATATATTTAAATTACATTTTTTTATATTCAGAATATAACTAAAAAGTAAATA 4560
AAAGAATTACATGAAAATTATCGTAGCGGCGTC**GAAACGAACAAAGAAACCTG**CGGTTGCGGCAGCGGCTGCGGCAGCGT 4640
CTGCGGCAACAAAACGAACAACAACCTGCGGTAGTGTTTGCCGCCGCCGCTGCCGCAACCGCCCAGGCGTAATGAAACGA 4720
ACAGGCTAATTTATATGTGTGAAGACTGAAAAAAAGTGATTTCGTCAAATGTAAAACCATAATTCTTGGCACTTCCTTAA 4800
TCGTTGTTATCAGCCATAGCGAATGTCTATTGCAATCTCATTCTCAAGTGTATGAGGTTATTTAAATCTTATTACTTAAG 4880
AAAAAAGGTTTGTTTCAAAGTTTTGATGTCTTATGTTATGTTGTTTTATTCCTTGTTTAAAACACATAAGTTAACAAGAA 4960
ATATACGAAAGAAAAAGCTATAACATCTGTCGCAAAACACTAACAATCCCAATAACATTTAGAACCAAATGATATAGGAT 5040
ACAAATAAACTCAAAACTTAATTTCTTCAAGTCAACAAAATTACGTATAAATATTGTGAAAATTACTCAGACTGCACTCT 5120
ACTCTTTGCAAAAATAACTCATTTAACACCTCATGGAAAATGTTTACTAGAGTAACCCAAATCTTTAGATTTTACAATAA 5200
CTGTAAAATGATTTTAAATGATTTTTTAGATTTTATATATAAAAAAAAGTGATTCAGGGTTTGATTTTATAATTAAATGC 5280
GTTTAATTCTACTTTTTTAattagcCgAtAgAtTCTgaTTgaTAGAAAAAAAAAAGAGTGAATGAATATTTTAAATATAA 5360
TTAGATATTTGCATGGGATTGAAAATTTGAGGAGGTTTTCAACAATTGAAATCATGGTCGTGAACAATGTTTATCTTTAG 5440
TTGGAGGTCTCCCTCTAAGCGCTAGTCATGAGTTGAGCTTGATGTGTTTTATGAACATCATTCTTTATTTTGGACATTCT 5520
GATGATTTTTATTTATTTATTTTCCTTAGTAATATTTCGCAAAATATGGGTTTTGGATCGGAGAAATTTTAACTTTCGTA 5600
AAGTTTAAATTGCATGTTATGGAACTAATCTTGTTGAGGTGTTGTGCGTCACCTAGAAACTTCTGGAACCTCGAGACTTG 5680
GTTAGATATGATTTATATAAGTACGTTTAAACTATTAGTGTATCCATATATAAGAAACCCAACAGTAGTGGAGTTGACAG 5760
TTCAATATTGATAGTCAGATGTAGTTGCTGGAATTTGCAAACTTAGATCAATGAACACTATAATGCACGAGTAGACAATC 5840
ATCAATATATTATCAATACAAGATTCACATATATCATAACATCTCTTGATAACATTATTACATTAACAAAAACAGACGGA 5920
TGGATCAAAA***CCCAAACACACATACACAAACTC***TAAGGGACAATATAATGAATCAAAAGTAACGTAGCACTATTTATATA 6000
TACAACAACAGAATTGAAACTTGATAATGTTTAATACGAACATGAATTCAACTTATTTTCAGCACATTCTTCGATTTAT 6080
TATGAAAAAACGAAACCAGCTCAACATAACCCATTGAAACATTTCATGAAATACCTGGCAGGCAATACTTGGTAGCAATG 6160
CCTTTGTGTTTAAAGTTTGGTTTGAGAGGACCACTGTGGAAGAAATGTCTTGGGCAGAGTCGAGTTGGGTTTGACCTGAA 6240
GAAAGAGAAGAAGAGTCGTTATATATAAAGAGATCTTTGGATCTTCTTTAGTTCTTTGGCCACTTCCACCATCGTTGGAA 6320
CTTCTTCACTAAGGCCGGTGCATCTCAGTGATAGCATCATGAAAGCTTTCATTTGACAACGCTCTTGTTCTGAAATTTGA 6400
CCCATCATTTCTAGCATCTTTGGATCTGCAATCTCTTCCATTGGTCTGTCTTCCTTTAATGTTGAGAGCAAATTCCGAGC 6480
ATGTCTTTTCATCTTACTGTTAAATTCATCTTCACTTTCTTCTCCTTTTCCTCGATAATGCTCGAAAAAGCTTTCGTTTC 6560
CTAATAGAAGCCTATGACCCATAAACATTCCAAAGGCAAAGACATCTGTTTTATCTGAGACTACGCCACTGCTCAAGTAA 6640
TTATCGTCCAAGAAACTATATATTC**CCTCTGCTGGTCTCTCAACC**CGGACAAATGTTTCTCCTTCTGGTATTGAGACGCA 6720
GTGAGAGAAATCAGTCAGCTTGGCGACACCATCTTCATCCAATAAGATATTCCAAAGAGACAAAATCCTATATACCAAGG 6800
GCCTAGGGAAGGCGGTGTGAAGGTAAGCTAAAGCAGTAGCGATATCTTCTGCTATCTTCATTCTCCTTTTCCATGGCTGT 6880
TCACTTATTTCTAATTTGAAATGTTTCTTAACACCATGATAGACTAAGACTGGATCTTCAAACTCAAGACAACATCCAAC 6960
CAATTTCATAAAGTTTTTGTGACCACTCACCATCGATGCAACTGCTATGTCGCGACACACTAAACTATCTACTCTCCTGC 7040
CATTCCA**CCAGCTAATACCGTTCCTGATGAGTATCAT**GGGATGGTTCTCGTTCTTACCTGAATACCATTGGTAGGCAAAA 7120
TCTTCTCGATAAACAAGATTAGAGTCGCTGAAATTGTCGGTGGCTTTGCGGATCTCATCAGCAGAGAAGAATTTAATGGG 7200
ATTGGATTTGCCATGGCCGAATTCGATCAGATCTTTTAACAAGATGCCT**CCTCTCTCCAATATTAGCTT**CTTATTCCTCT 7280
TCCTCCACCACCAACCCATCTCTAAACCTAAACTCTTTTTTTTCTGTTTTTTTTTATAAAAGAAAGACTTTAGTTTTAAA 7360
TCAAATGAGCAGAGAGGTTTTTCAAGAAATGAGGTTTTTCATAATGAGTTTGCAAACGAAATGGATAATAAGATTAGATT 7440
TTTAAGGTACTCAAATTCTTGTTGAA***TTTTCAACATTGACGTCCTCC***CGCGAAAATTCAGAAATAAACATTTTAATTGTT 7520
TTTGTGGAATTTAAAATTGGAAGGATTTATCACATATCTTGGTGATCTCTACGATTTTGGGGTCTACTCATGTCATCAGT 7600
TTCTTTTTTTATAAAAAAATATCTTTTTACTTGTTAGTGAAGAACGGTCTTCAAGAATTCAAACACTCGGCTGGAATTAG 7680
ATCATCTAGTGTATAAATTAATTGTTAAGATTCTTTCAAGCTAGCTGTCCAGTTACGCAAATTGTTCGTAAATCAGATTC 7760
TTTTTTTAACCTAAATCATCACCCAATTTGTTCTTATTATACACTTCTTGTAATATTTTACTAAAACTAAGAATTTTGTA 7840
CAAGTGGCTAAGCTTTCAATTCTTGAGACTTCTACAGCTTTGAGACTTCCAAATCGCTAGACATCGAATGAGAGATGTTG 7920
CACCAACTTCTCCAGTTTACTATCAATCATAGATGACCGCAGAAAAAAAA 7970
