# Supplementary material for: ZED1-related kinase 13 is required for resistance against Pseudoidium neolycopersici in Arabidopsis accession Bla-6
Source: Front Plant Sci. 2023 Mar 21;14:1111322. doi: 10.3389/fpls.2023.1111322 (PMC10071312; doi:10.3389/fpls.2023.1111322)
Supplement: Supplementary file 12 [file Image_7.pdf]

Supplementary Figure 7. Pairwise alignment of the genomic sequence of *ZRK13* of Col-0 and Bla-6. Primers for sequencing are indicated in blue rectangles, start codon in green rectangle, and stop codons in red rectangles.

```
                                                 LZRK13Fw
Col-0   AAACAATTAAAATGTTTATTTCTGAATTTTCGCGGGAGGACGTCAATGTTGAAAATTCAA     60
Bla-6   AAACAATTAAAATGTTTATTTCTGAATTTTCGCGGGAGGACGTCAATGTTGAAAATTCAA     60

Col-0   CAAGAATTTGAGTACCTTAAAAATCTAATCTTATTATCCATTTCGTTTGCAAACTCGTTA    120
Bla-6   CAAGAATTTGAGTACCTTAAAAATCTAATCTTATTATCCATTTCGTTTGCAAACTCATTA    120

Col-0   TGGAG--------TTCTTGAAAATACTCTCTGCTCATTTGATTTAAAACTAAAGTCTTTC    172
Bla-6   TGAAAAACCTCATTTCTTGAAAAACCTCTCTGCTCATTTGATTTAAAACTAAAGTCTTTC    180
                                                    start codon
Col-0   TTTTATAAAAAAAAAAAACAGAAAAAAAAACAGTTTAGGTTTAGAGATGGGTTGGTTGTG    232
Bla-6   TTTTATAAAAAAAAA---CAGAAAAAAAA-GAGTTTAGGTTTAGAGATGGGTTGGTGGTG    236

Col-0   GAGGAAGAAGAAAAAGAATAAGAAGCTAGAATTGGAGAGAGGAGCCAAGTTGTTAGAAGA    292
Bla-6   GAGGAAG------AGGAATAAGAAGCTAATATTGGAGAGAGGAGGCATCTTGTTAAAAGA    290

Col-0   GCTCATCGAATGTTGCGATGGCAAATCCAATCCCATCAAATTCTTCTCTGCTGATGAGAT    352
Bla-6   TCTGATCGAATTCGGCCATGGCAAATCCAATCCCATTAAATTCTTCTCTGCTGATGAGAT    350

Col-0   CCGCAAAGCCACCAACATTTTCAGCCACTCTAATCTTGTTCATCAGGAAGAATTTTACTG    412
Bla-6   CCGCAAAGCCACCGACAATTTCAGCGACTCTAATCTTGTTTATCGAGAAGATTTTGCCTA    410

Col-0   CCAATGGTATTCAGGTAAGAACGAGAACCATCCCATGATACTCATCAGGAAAGATTCT--    470
Bla-6   CCAATGGTATTCAGGTAAGAACGAGAACCATCCCATGATACTCATCAGGAACGGTATTAG    470

Col-0   -------AATGTGAGGGGAGGAGA---TCTTGTGTGCCGCAACATAGCAGTTTCATCGAT    520
Bla-6   CTGGTGGAATGGCAGGAGAGTAGATAGTTTAGTGTGTCGCGACATAGCAGTTGCATCGAT    530

Col-0   GGTGAGTGGTCACAAGAACTTTATGAAATTGGTTGGATGTTGTCTTGAGTTGAAATATCC    580
Bla-6   GGTGAGTGGTCACAAAAACTTTATGAAATTGGTTGGATGTTGTCTTGAGTTTGAAGATCC    590

Col-0   AGTCACGGTCTATCATGGTGTTAAGAAACATTATGGATTAGAAATAGATGAGAAGCCGTG    640
Bla-6   AGTCTTAGTCTATCATGGTGTTAAGAAACATTTCAAATTAGAAATAAGTGAACAGCCATG    650

Col-0   GAAAAGAAGAATGAAGATAGCAGAAGATATCGCCACTGCTTTAGCTTACCTTCACACTGC    700
Bla-6   GAAAAGGAGAATGAAGATAGCAGAAGATATCGCTACTGCTTTAGCTTACCTTCACACCGC    710

Col-0   CTTCCCTAGGCCCCTCGTATATATGATTTTGTCTCATCGGAATATCTTATTGGATGAAGA    760
Bla-6   CTTCCCTAGGCCCTTGGTATATAGGATTTTGTCTCTTTGGAATATCTTATTGGATGAAGA    770

Col-0   TGGTGTCGCGAAGCTGACTGATTTCTCTCACTGCGTCTCAATCCCAGAAGGAGAAACATT    820
Bla-6   TGGTGTCGCCAAGCTGACTGATTTCTCTCACTGCGTCTCAATACCAGAAGGAGAAACATT    830

Col-0   TGTCCGGGTTGAGG---CAGAAGATGGATTCTATAGTTACTTTGCTGATAATTACGTTAA    877
Bla-6   TGTCCGGGTTGAGAGACCAGCAGAGGGAATATATAGTTTCTTGGACGATAATTACTTGAG    890

Col-0   CAGTGTTGTAGTCTCAGAGAAAACAGATGTCTTTGCCTTTGGAATCTTTATGGGTCTAAC    937
Bla-6   CAGTGGCGTAGTCTCAGATAAAACAGATGTCTTTGCCTTTGGAATGTTTATGGGTCATAG    950

Col-0   GCTTCTATTAGGATACAAAAGCTATTTCGAGCATTATCGAGGAGAAGAAAAAGAAAGTGA    997
Bla-6   GCTTCTATTAGGAAACGAAAGCTTTTTCGAGCATTATCGAGGAAAAGGAGAAGAAAGTGA   1010

Col-0   AGAAGAAGACCCTGAAGATACTGATGAATTAGATTATTTTAACAAGAAAAGACATGCTCG   1057
Bla-6   A---------------------GATGAATT---TAACAGTAAGATGAAAAGACATGCTCG   1046

Col-0   ATATTGGCTGTCAAAATTAAAGAAAGACCGACCAATGGAAGAGATTGCAGATCGAAAGAT   1117
Bla-6   GAATTTGCTCTCAACATTAAAGGAAGACAGACCAATGGAAGAGATTGCAGATCCAAAGAT   1106
```

```
Col-0   GATAAAAAATATGGGTCAAATCTTAGAACAAGAGCTCTTTCAAATGAAAGCTTTCCGGAT   1177
Bla-6   GCTAGAAATGATGGGTCAAATTTCAGAACAAGAGCGTTGTCAAATGAAAGCTTTCATGAT   1166

Col-0   GCTCTCACTGAGATGCATGGGTCCTAGTGAAGAAGTTCCAACGATGGTGGAGGTGGCCAA   1237
Bla-6   GCTATCACTGAGATGCACCGGCCTTAGTGAAGAAGTTCCAACGATGGTGGAAGTGGCCAA   1226

Col-0   AGAACTAAAGAAGATCCAAAAATCTCTTAATAAAGATTCTTATCGAGGAGAAGAAGAAGA   1297
Bla-6   AGAACTAAAGAAGATCCAAAGATCTCTTTAT-----------------------------   1257

                                 stop codon Bla-6
Col-0   AGAAGAAAGTGAAGATGAATTTAACGACTCTTCTTCTCTTTCTTCAGGTCAAACCCAATT   1357
Bla-6   ------------------ATATAACGACTCTTCTTCTCTTTCTTCAGGTCAAACCCAACT   1299

                                                          stop codon Col-0
Col-0   CGACTCTGCCCAAGACATTTCTTCCACAGTGGTCCTCTCAAACCAAAC-TTAAACACAAA   1416
Bla-6   CGACTCTGCCCAAGACATTTCTTCCACAGTGGTCCTCTCAAACCAAACTTTAAACACAAA   1359

Col-0   GGCATTGCTACCAAGTATTGCCTGCCAGGTATTTCGTGAAATGTTTCAATGGGTTATGTT   1476
Bla-6   GGCATTGCTACCAAGTATTGCCTGCCAGGTATTTCATGAAATGTTTCAATGGGTTATGTT   1419

Col-0   GAGCTGGTTTCGTTTTTTCAGGAAAAATCGAACAATGTGCTGAAAATAAGTTGAATTCAT   1536
Bla-6   GAGCTGGTTTCGTTTTTTCATAATAAATCGAAGAATGTGCTGAAAATAAGTTGAATTCAT   1479

Col-0   GTTCGTATTAAAACATTATCAAGTTTCATTTTTGTTGTTGTATTTATAAATAGTGCTACG   1596
Bla-6   GTTCGTATTAAAACATTATCAAGTTTCAATTCTGTTGTTGTATATATAAATAGTGCTACG   1539

                                              LZRK13Rv
Col-0   TTACTTTTGATTCATTATATTGTCCCTTAAGAGTTTGTGTATGTGTGTTTGGGTTTTGAT   1656
Bla-6   TTACTTTTGATTCATTATATTGTCCCTT-AGAGTTTGTGTATGTGTGTTTGGGTTTTGAT   1598

Col-0   CGATCCGTCTGTTTTTGTTAATGTAATAATTTTATCAAGAGATGTTATGATATATGTGAA   1716
Bla-6   CCATCCGTCTGTTTTTGTTAATGTAATAATGTTATCAAGAGATGTTATGATATATGTGAA   1658

Col-0   TCTTG-----------TATTGATGATTGTCTACTCGTGTATTATA                    1750
Bla-6   TCTTGTATTGATAATATATTGATGATTGTCTACTCGTGCATTATA                    1703
```
